# Genetic structure, phylogeography, and demography of *Anadara tuberculosa* (Bivalvia) from East Pacific as revealed by mtDNA: Implications to conservation

**DOI:** 10.1002/ece3.4937

**Published:** 2019-04-04

**Authors:** Benoit Diringer, Krizia Pretell, Ricardo Avellan, Cesar Chanta, Virna Cedeño, Gabriele Gentile

**Affiliations:** ^1^ Incabiotec Tumbes Peru; ^2^ Universidad Nacional de Tumbes Tumbes Peru; ^3^ Cienciactiva‐Concytec Lima Peru; ^4^ Concepto Azul Guayaquil Ecuador; ^5^ Department of Biology University of Rome Tor Vergata Rome Italy

**Keywords:** aquaculture, aquatic animal stock, last glacial maximum, mangrove habitat, mollusk, oceanic current

## Abstract

Wild populations of the pustulose ark, *Anadara tuberculosa* (Bivalvia), an emblematic species of the East Pacific mangrove ecosystem declined in South American countries (Colombia, Ecuador, and Peru) mainly due to overharvesting and habitat loss or degradation. Understanding the genetic aspects of geographic variations and population structure of *A. tuberculosa*, currently unknown, appears as a priority to fishery authorities in order to elaborate integrated and collaborative conservation policies for fishery management, aquaculture, and stock enhancement programs. We used mtDNA sequence data to investigate haplotype diversity, genetic structure, and demography of *A. tuberculosa*. Results indicate genetic homogeneity of populations distributed north and south of the equator, respectively. However, statistically significant differentiation emerged between northern and southern populations with pairwise *ф*_ST_ values ranging between 0.036 and 0.092. The oceanic current system acting in the area (Panama Current and Humboldt Current) might play a role in limiting the larval dispersal of the species, still poorly understood. Demography reconstruction supported recent population expansion, possibly started after last glacial maximum. Our results would suggest separate and independent management of populations north and south of the equator.

## 1 INTRODUCTION

The pustulose ark *Anadara tuberculosa* (Mollusca, Bivalvia) is distributed from the Gulf of California in Mexico to the region of Tumbes in northern Peru (Baqueiro, Massó, & Guajardo, 1982). Natural banks of this species develop in muddy sediments, in particular around the roots of the red mangrove, *Rhizophora mangle* (Camacho, 1999). Limited information exists on the biology of the species. Available data indicate variability in life history traits (Flores, Licandeo, Cubillos, & Mora, 2014). Reproduction may occur during the whole year, although massive spawning may exist locally, usually correlated with higher peaks of food availability (Garcia Dominguez, De Haro‐Hernandez, Garcia‐Cuellar, Villalejo, & Rodriguez‐Astudillo, 2008; Lucero, Cantera, & Neira, 2012). This ark is a symbolic species of the East Pacific mangrove ecosystem that has been ancestrally collected by coastal populations as a staple food and that is still consumed for traditional meals in several tropical Latin American countries. Extraction is an essential activity for numerous families whose economy relies on *A. tuberculosa* trade. Most of the natural stocks of *A. tuberculosa* are over exploited, and some populations are close to collapse in several countries (Lucero et al., 2012; Mora, Moreno, & Jurado, 2011). In Peru, the *A. tuberculosa* population of Tumbes Region has been reduced by 6.4‐fold between 1988 and 2008 in unprotected mangrove areas as well as in the protected National Mangrove Sanctuary of Tumbes (SNLMT) (Ordinola, Montero, Alemán, & Llanos, 2010; Vivar, 1996). The diminution of *A. tuberculosa* population, like the majority of bivalve natural stocks worldwide, results from several factors such as overexploitation, habitat degradation, and nonidentified mortalities.

To recover depleted aquatic animal stocks, strategies generally rely on quota implementation with, on one hand, permanent collection restrictions based on animal minimum size limits and, on the other hand, occasional collection prohibitions during reproductive seasons. Such regulations are more or less respected and are complicated by their economic consequences for fishermen. Another strategy component for recovering aquatic animal stocks is based on habitat restoration (McCay, Peterson, De Alteris, & Catena, 2003).

Strategy for restocking and enhancing stocks of natural aquatic populations has also been focused on the mass release of hatchery‐produced animals (Arnold, 2008; Bell, Rothlisberg, & Munro, 2005). Such a strategy is attractive for aquatic species, in particular mollusks, considering their extremely high fecundity and the subsequent possibility to produce in hatchery millions of spat from a numerically limited wild broodstock. In fact, aquaculture has started in El Salvador and Costarica (FAO, 2017). However, such a strategy could also lead to a quick reduction of population genetic diversity, generating negative effects. Within this context, understanding the genetic aspects of geographic variations and population structure of *A. tuberculosa* appears as a priority to fishery authorities in order to elaborate integrated and collaborative conservation policies for fishery management, aquaculture, and stock enhancement programs in a concerted way.

At present, genetic studies of *A. tuberculosa* still lack and no information is available about the genetic structure of the species at a large geographic scale. In particular, gaining insight on the possible role of marine equatorial currents in shaping the genetic structure of the South American populations could prove very useful for the identification of sanctuaries aimed at the preservation of the genetic variation of the species. In this study, we used mtDNA sequence data (partial COI gene) to investigate haplotype diversity, genetic structure, and demography of *A. tuberculosa* from the southern edge of its geographic distribution. Samples were collected from two sampling sites north of the equator (Colombia and Ecuador) and three south of the equator (Ecuador and Peru).

## 2 MATERIALS AND METHODS

### 2.1 Sampling

Samples of *A. tuberculosa* were collected in Peru: National Sanctuary Mangrove of Tumbes (3°25′31.56′′S, 80°16′30.39′′W) in 2011; Ecuador: El Oro (2°21′05.27′′S, 80°14′52.03′′W), Guayas (2°49′55.60′′S, 80°7′44.05′′W), Esmeraldas (1°17′08.79′′S, 78°47′41.79′′W) in 2015; Colombia: Tumaco (2°30'40.4"N, 78°29'40.3"W) in 2016. Two hundred forty‐two individuals were sampled in total. Sample size for each sampled locality is reported in Table 1.

**Table 1:** Genetic diversity in *Anadara tuberculosa*

	Sample size	Haplotype diversity (*h*)	Nucleotide diversity (*π*)	No. of haplotypes	No. of private haplotypes
Tumaco (Colombia)	50	0.962 ± 0.019	0.008 ± 0.0001	23	10
	*24*	*0.888 ± 0.046*	*0.007 ± 0.0009*	*12*	*7*
Esmeralda (Ecuador)	29	0.874 ± 0.037	0.006 ± 0.0007	11	4
	*24*	*0.855 ± 0.048*	*0.006 ± 0.0008*	*10*	*3*
Guayas (Ecuador)	24	0.870 ± 0.055	0.005 ± 0.001	12	2
	–	–	–	–	*4*
El Oro (Ecuador)	28	0.987 ± 0.014	0.008 ± 0.0007	24	11
	*24*	*0.986 ± 0.018*	*0.008 ± 0.0008*	*21*	*11*
Tumbes (Peru)	111	0.898 ± 0.021	0.006 ± 0.0005	39	20
	*24*	*0.895 ± 0.058*	*0.006 ± 0.001*	*16*	*8*

Note

Estimates obtained after randomly resampling 24 individuals from each sample are in italic.

### 2.2 DNA extraction and amplification

Total genomic DNA was individually extracted from approximately 100 mg of tissue that could be spats, gill, and mantle, using a standard CTAB protocol (Folmer, Black, Hoeh, & Lutz., & Vrijenhoek, 1994).

Primers used were as proposed by Folmer et al. (1994), to obtain a partial sequence (620 bp) of the COI mitochondrial DNA gene. PCRs were performed in a total volume of 50 µl including 1.5 U High fidelity Platinium Taq DNA polymerase (Invitrogen), 100 ng of template DNA, 20 pmol of forward and reverse primers (LCO 1490 and HCO 2198), 0.2 mM of each dNTP and 1X PCR buffer, and 1.5 mM of MgCl2. The PCR was carried out under the following conditions: an initial denaturation for 5 min at 95°C, then 35 cycles of denaturation for 30 s at 94°C, annealing for 45 s at 50°C, followed by extension for 1 min at 72°C and a final extension step of 7 min at 72°C. PCR amplicons were sent to sequencing at MACROGEN (USA).

### 2.3 Data analyses

Sequences were edited with the software Mega5 (Tamura, Stecher, Peterson, Filipski, & Kumar, 2013) and aligned by Muscle (Edgar, 2004).

Haplotype and nucleotide diversities were estimated by using DnaSP ver. 6.10.01 (Librado & Rozas, 2009). Because the number of haplotypes and private haplotypes depended of sample size (Supporting Information Figure S1), we also recalculated all statistics after randomly extracting 24 individuals from each population, to match the minimum sample size. Among‐ and within‐group *F*_ST_, *F*_SC_, *ф*_ST_, *ф*_SC_, and pairwise *F*_ST_ and *ф*_ST_ estimates were calculated by using Arlequin ver 3.5.2.2 (Excoffier & Lischer, 2010). The same software was used to perform Mantel tests (Mantel, 1967). The tests were run to investigate possible isolation by distance (IBD) by estimating correlation between the matrix of pairwise geographic distances between sampling locations and two correspondent matrices of estimates of genetic differentiation: *F*_ST_, based on haplotype frequency, and *ф*_ST_, that takes into account genetic distances among haplotypes (Bird, Karl, Smouse, & Toonen, 2011). Geographic distances were calculated as the shortest pathways along the costal line.

Genealogical relationships among different haplotypes were investigated by applying maximum parsimony (Templeton, Crandall, & Sing, 1992) as implemented in TCS software (Clement, Posada, & Crandall, 2000).

Demographic histories were studied using DnaSP ver. 6.10.01 to estimate three different classes of statistics under the assumption of neutrality. We estimated class I D*, F* (Fu & Li, 1993), and D (Tajima, 1989) test statistics, which use information of the mutation frequency (segregating sites). We also estimated Fs (Fu, 1997), which uses information from the haplotype distribution (class II). Finally, we calculated Harpending's raggedness (*r*) index (Harpending, 1994), based on the distribution of the observed pairwise nucleotide site differences (mismatch distribution, class III), and the expected values in populations with constant population size and in growing populations (Rogers & Harpending, 1992). Such statistic is expected to show lower values in mismatch distributions of expanded populations but has little power to detect population expansions. For all statistics, we used the coalescent algorithm implemented in DnaSP to estimate the probability of obtaining values that, under the tested demographic model, would be lower than the observed (one‐tailed test).

Following a different approach, different demographic models (constant population size, exponential growth, and Bayesian skyline ‐ BSP) were also investigated using Beast 2.4.5 (Bouckaert et al., 2014). Because the BSP makes few a priori assumptions about the historical demographic trend of the population, it can guide to formulate more specific demographic hypotheses (Crandall, Sbrocco, DeBoer, Barber, & Carpenter, 2012). We used the software jModeltest 2.1.7 (Darriba, Taboada, Doallo, & Posada, 2012) and selected the TIM3+I+G substitution model (AC = CG, AT = GT; unequal base frequencies, invariant sites and gamma distribution), based on the Akaike Information Criterion (Akaike, 1973). For both constant and exponential growth models, a lognormal prior was selected as coalescent population size parameter and a strict clock model was assumed for all Beast analyses. Runs for all analyses consisted in 100 million MCMC iterations with sampling every 1,000 and a 10% burnin. Convergence was considered reached when the effective sample sizes >200. Demographic models were tested by estimating Bayes factors for all pairwise model comparisons. Bayes factors were calculated by using marginal likelihood natural logarithm estimated by path sampling calculation (Baele et al., 2012). We established 15 steps for the proper estimation of marginal likelihood. In fact, around this value the marginal likelihood estimate remained constant. Runs for marginal likelihood estimation consisted in 50 million MCMC iterations with sampling every 1,000 and a 50% burnin. The Bayes factor for each pairwise model comparison was estimated as the difference of the marginal likelihoods of the two compared models. If this difference was positive then the Bayes factor was in favor of model 1; if it was negative, it was in favor of model 2. Aware that population structure may have a confounding effect on Bayesian skyline plot inferences of demographic histories (Heller, Chikhi, & Siegismund, 2013); we performed all demographic analyses by grouping populations according to a criterion as in Buonaccorsi et al. (2004). We therefore considered the best grouping methods those in which among‐group heterogeneity was maximized (largest and significant *F*_ST_ or *ф*_ST _estimates) and within‐group heterogeneity minimized (smallest and least significant *F*_SC_ or *ф*_SC _estimates). In this case, the analysis was restricted to adjacent groupings, under the implicit assumption of possible IBD. Further support was also sought by comparing pairwise *F*_ST_ and *ф*_ST_ estimates.

In the absence of prior information on time of divergence, the Bayesian demographic reconstruction as implemented in Beast returns time estimates in units of mutations/site. To be translated in years, such time estimation must be divided by a mutation (substitution) rate (μ), usually expressed in units of percent change per million years (%/Myr). We here consider two COI lineage mutation rates (1/2 divergence rate) for marine invertebrates as proposed by Crandall et al. (2012) who estimated molecular rates to exponentially decay from 5.3%/Myr (to be considered as an instantaneous mutation rate after lethal mutations have been removed) to a long‐term rate of 0.65%/Myr. Whereas the second rate is certainly too low to effectively trace recent events, the first one might not sufficiently account for purifying selection that keeps removing variation even after lethal mutations have been eliminated. However, if divergence is recent, purifying selection is not expected to have played a major role yet; therefore, a high mutation rate is conceivable.

### 2.4 Oceanic surface currents

**Figure 1:**
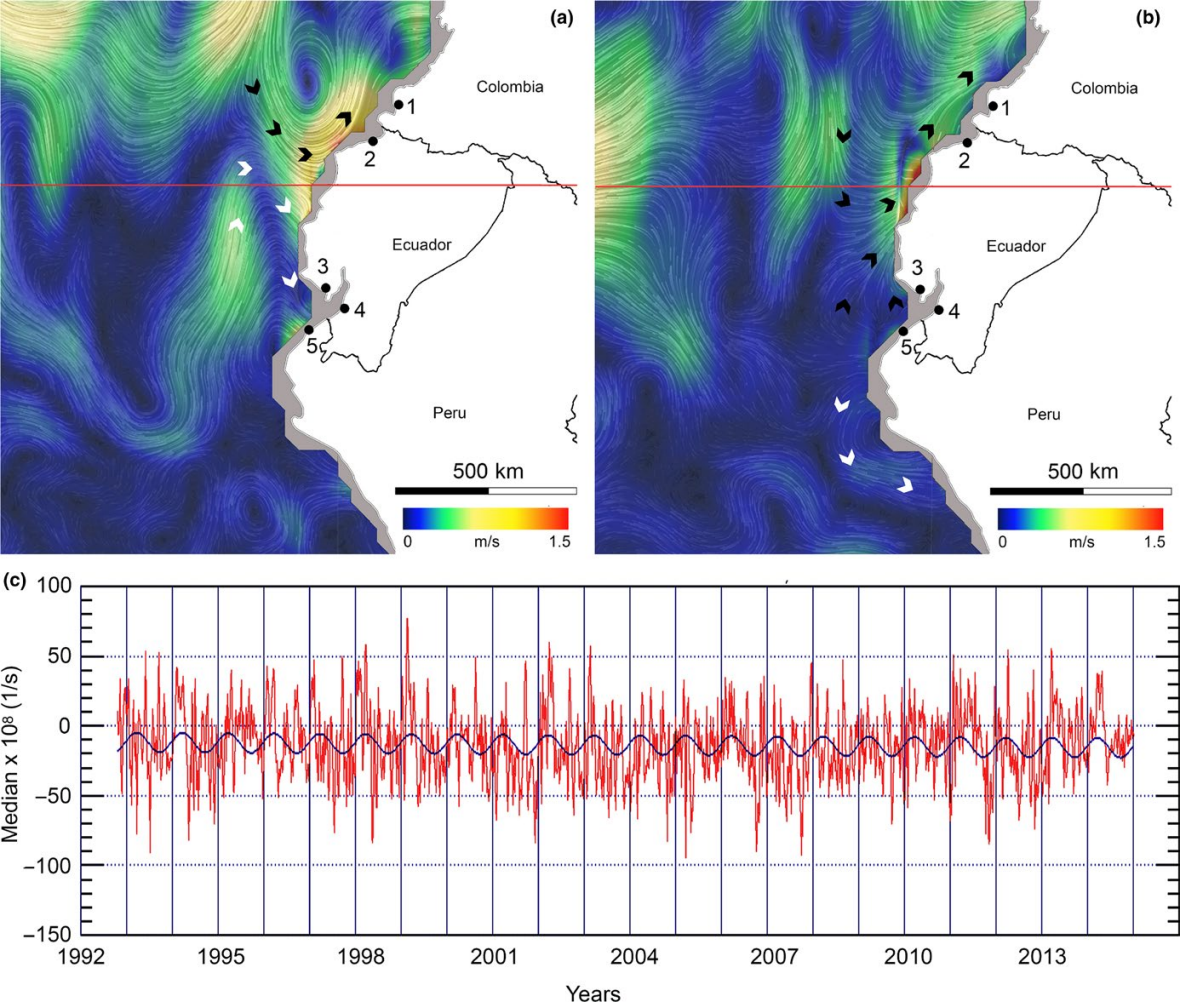
Sampling sites: 1) Tumaco (Colombia), 2) Esmeralda, 3) Guayas, 4) El Oro (Ecuador), 5) Tumbes (Peru). Surface oceanic circulation observed in February 2018 (a) and March 2018 (b). Current convergence (periodic median values) in the equatorial region comprised between latitude 1.8 S and 0.2 N and longitude 82.2 W and 80.2 W, from 1992 to 2015 (c). The red line indicates the equator. The overall pattern of circulation, although with some variation, tends to be stable over time

A map of oceanic surface currents has been obtained from https://earth.nullschool.net/about.html, copyright 2018 Cameron Beccario) which uses data from the program Ocean Surface Current Analyses Real‐time (OSCAR, Earth & Space Research, https://www.esr.org/research/oscar/oscar-surface-currents/) (Figure 1). Such map reports surface oceanic circulation observed in February 2018 (a) and March 2018 (b). Data of surface currents convergence were obtained from http://oceanmotion.org/html/resources/oscar.htm#visstart, also based on OSCAR data. Current convergence measures how strongly the current flows toward or flows away from a location. Whereas a positive convergence of water is evidence of downwelling, a negative convergence indicates upwelling. Figure 1c reports the median value of convergence in a region comprised between latitude 1.8 S and 0.2 N and longitude 82.2 W and 80.2 W, from 1992 to 2015.

## 3 RESULTS

### 3.1 Genetic variation and differentiation

Overall, 69 different haplotypes were found. The number of haplotypes decreased to 47 after reducing sample size throughout sampling sites (*N* = 120, see Table 1). Haplotype diversity (*h*) ranged between 0.874 (±0.037, *SD*) and 0.986 (±0.018, *SD*). Nucleotide diversity (*π*) ranged between 0.005 (±0.001, *SD*) and 0.008 (±0.001, *SD*). The number of haplotypes and private haplotypes appeared positively and linearly correlated to sample size (Supporting Information Figure S1). Haplotype and nucleotide diversities were not strongly affected by the reduction of sample size, which in turn strongly influenced both the number of haplotypes and private haplotypes (Table 1).

Values of *F*_ST,_ *ф*_ST,_ *F*_SC,_ and *ф*_SC _associated to different assemblages of populations in 2, 3, and 4 groups are reported in Table 2. Assemblages B (two groups: Tumaco‐Esmeralda/Guayas‐El Oro‐Tumbes) and E (three groups: Tumaco/Esmeralda/Guayas‐El Oro‐Tumbes) showed largest, highly statistically significant *F*_ST _and *ф*_ST _and correspondent lowest, not significant *F*_SC_ and *ф*_SC_.

**Table 2:**
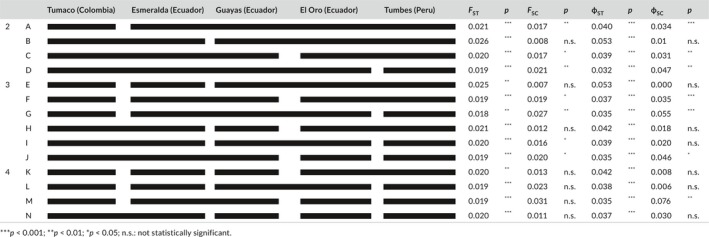
Population grouping. The first column indicates the number of groups considered. The second column denotes the combination of populations in each group, indicated by continuous black lines. We estimated the probability (*p*) that observed F_ST_ and ф_ST_ values are lower or equal to random values. We also estimated the probability that observed F_SC_ and ф_SC_ values are higher or equal to random values. Random values were obtained from 1000 random permuted samples

The pairwise *F*_ST_ and *ф_ST_* analysis (Table 3) also indicated that differentiation exists between sites north to the equator (Tumaco and Esmeralda) and those south to it (Guayas, El Oro, Tumbes), with significant *F*_ST_ and *ф_ST_* values ranging between 0.037 and 0.092 (*p *« 0.05). Given the combined evidence provided by the two analyses, we considered assemblage B for subsequent demographic analyses.

**Table 3:** Pairwise *F*_ST_ and *ф*_ST_ estimates (below and above the diagonal, respectively) with correspondent statistical significance

	Tumaco (Colombia)	Esmeralda (Ecuador)	Guayas (Ecuador)	El Oro (Ecuador)	Tumbes (Peru)
Tumaco (Colombia)	–	0.025 n.s.	0.039*	0.040*	0.036***
Esmeralda (Ecuador)	0.011 n.s.	–	0.092**	0.072**	0.087***
Guayas (Ecuador)	0.024*	0.034*	–	−0.011 n.s	0.000 n.s.
El Oro (Ecuador)	0.015*	0.026*	0.022 n.s.	–	0.009 n.s
Tumbes (Peru)	0.024**	0.033**	−0.004 n.s	0.012 n.s.	–

Note

n.s., not statistically significant.

*** *p* < 0.001.

** *p* < 0.01.

* *p* < 0.05.

The correlation coefficients resulted from Mantel test were *r*(*ф*_ST_) = 0.718, *p*(*r* rand >= *r* obs) = 0.097 and *r*(*F*_ST_) = 0.639, *p*(*r* rand >= *r* obs) = 0.092.

### 3.2 Haplotype network

**Figure 2:**
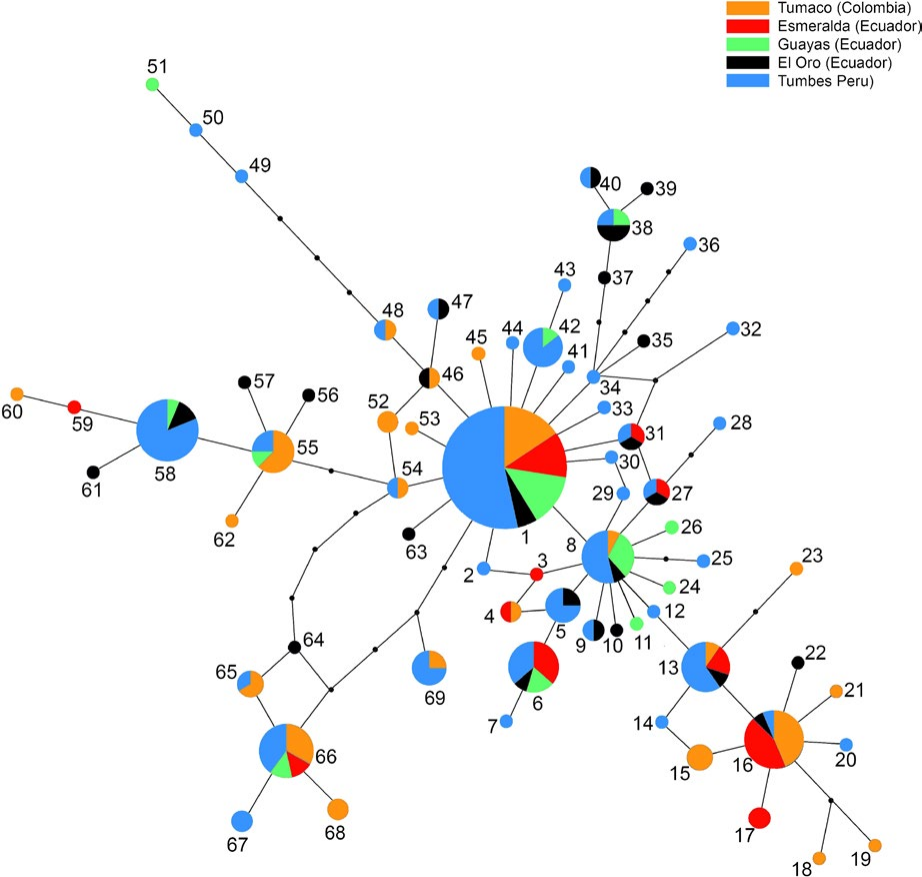
Statistical parsimony network illustrating the genealogical relationships among different haplotypes (threshold of statistical significance = 95%). The size of the circle corresponds to the haplotype frequency. Pie charts indicate the proportion at which each haplotype occurs at each location

Genealogical relationships between haplotypes are reported in Figure 2. Haplotype 1 showed the highest frequency in the sample and occurred also at all sites. Haplotype 8 occurred at almost all sites and is one step apart from Haplotype 1. Several singletons stemmed from Haplotypes 1 and 8, separated by a single substitution. Most of them were found at sites south to the equator. Longer branches departed from Haplotypes 1 and 8, some of them leading to groups of haplotypes geographically more localized at north or south to the equator. The unequal sample size biased the graph in favor of Tumbes (Peru), but did not alter the general topology of the network (Supporting Information Figure S2).

### 3.3 Demographic histories

**Figure 3:**
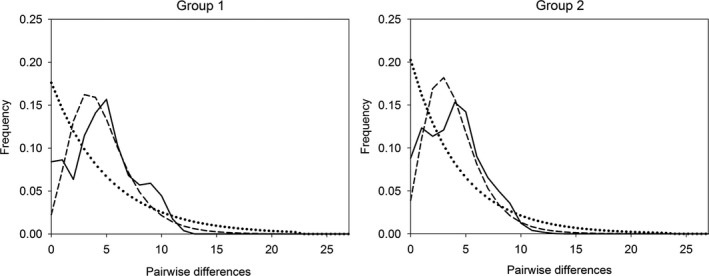
Mismatch distributions (pairwise differences) in Groups 1 and 2. Dotted and dashed lines indicate expected distributions under constant size and expansion models, respectively. Solid line indicates observed distributions

Based on *ф*_ST_ analysis, demographic histories were investigated by considering two groups of populations: Group 1 that included Tumaco and Esmeralda, and Group 2 that comprised Guayas, El Oro, and Tumbes. In all tests, D*, F*, Fs, and D were negative, but statistical significance was observed only for D*, F*, and D for Group 2 (D*: −3.051, *p* = 0.004; F*: −2,861, *p* = 0.003; D: −1.644, *p* = 0.022) and for Fs for both Groups 1 and 2 (Fs_Group1 _= −12.033, *p *« 0.001; Fs_Group2 _= −46.589, *p *« 0.001. Raggedness index were *r*_Group1 _= 0.009, *p* = 0.006 and *r*_Group2 _= 0.007; *p *« 0.001). For both groups, the observed mismatch distribution was more similar to the mismatch distribution expected under the population expansion model than under the constant size model (Figure 3). All summary statistics and their probability values are reported in Table 4.

**Table 4:** Demographic histories as inferred by different classes of statistics under the assumption of neutrality

Statistics	Group 1		Group 2	
	Obs	*p*(Sim <= Obs)	Obs	*p*(Sim <= Obs)
Tajima's D	−0.635	n.s	−1.644	*
Fu and Li's D*	−0.974	n.s	−3.051	**
Fu and Li's F*	−0.965	n.s	−2.862	**
Fu's Fs	−12.033	***	−46.589	***
Raggedness, *r*	0.009	**	0.007	**

Note

Group 1 includes Tumaco and Esmeralda, whereas Group 2 comprises Guayas, El Oro, and Tumbes.

n.s.: not statistically significant

*** *p* < 0.001

** *p* < 0.01

* *p* < 0.05

**Figure 4:**
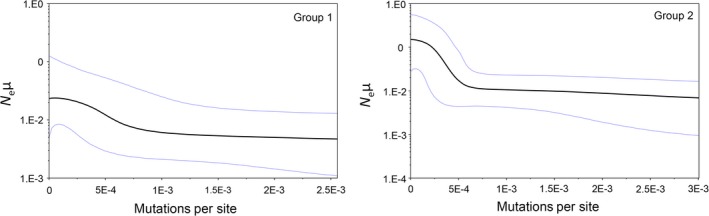
Bayesian skyline plots for Groups 1 and 2. *X*‐axis indicates time expressed in mutation per site units. *Y*‐axis indicates population size expressed as the product of *N*_e_ (effective population size) and μ (mutation rate)

The BSP (Figure 4) indicated a modest increase in N_e_μ for Group 1 (a). A clearer evidence of demographic expansion was shown by Group 2 (b). Bayes factors indicated the BSP as the best representation of the demographic history of Group 1, whereas the exponential model was preferred for Group 2 (Table 5).

**Table 5:** Demographic models comparison

Models 2	Models 1		
	Exp. growth	Constant size	BSP
Exp. growth	–	−5.79	−2.87
Constant size	−0.06	–	2.92
BSP	−9.47	−9.41	–

Note

Bayes factor for Group 1 (below the diagonal) and Group 2 (above the diagonal). BSP is selected for Group 1 and exponential growth for Group 2. Group 1 includes Tumaco and Esmeralda, whereas Group 2 comprises Guayas, El Oro, and Tumbes

## 4 DISCUSSION

### 4.1 Geographic variation

Our study showed high level of genetic variation in equatorial *A. tuberculosa*, as estimated using maternally inherited COI sequences. Haplotype diversity (*h*) and nucleotide diversity (*π*) were comparable to or higher than values observed in other natural populations of bivalves (Xue, Wang, Zhang, & Liu, 2014). Despite the potentially high larval dispersal, the species appears significantly structured and two groups of populations seem to exist, which comprise populations at north and south of the equator, respectively. A combination of life history traits and the pattern of oceanic and coastal current circulation in the equatorial east Pacific Ocean can provide a suitable explanation for the incomplete connectivity between Groups 1 and 2. In fact, fertilized eggs of *A. tuberculosa* rapidly develop into trochophore planktonic larvae. Such larvae undergo further changes until, after at least 21 days, they settle down in the bottom (Diringer, Vasquez, Moreno, Pretell, & Sahuquet, 2012). The speed of marine costal currents aside the equator can reach 1–1.5 m/s and water moves anticlockwise at north of the equator. The pattern of marine currents south of the equator is less stable, with coastal waters moving southward or northward in different time of the year (Figure 1a,b). Given the speed of currents and the time before settling, the maximum dispersal of a trochophore larvae could ranges between 1,000 and 2000 km. This could potentially allow gene flow between locations, with the south–north direction being predominant, as guided by the prevalent pattern of coastal currents. Despite IBD could not be completely ruled out because of the lack of sampling locations at intermediate geographic distances that would allow a proper test (see Supporting Information Figure S3), the north‐equatorial anticlockwise circulation could play an important role in reducing bidirectional gene flow. Furthermore, the east Pacific equatorial coast of South America shows a negative surface current convergence that indicates upwelling and outward flow. Overall, such conditions may impact on gene flow by subtracting larvae from coastal waters and exposing them to more oceanic, possibly unfavorable, conditions. Pelagic larval duration and pattern of oceanic currents proved very important in shaping geographic patterns of genetic variation in mollusks (Claremont, Williams, Barraclough, & Reid, 2011; Selkoe & Toonen, 2011).

### 4.2 Demographic histories

None of the demographic analyses performed supported constant size populations, although they depicted different scenarios for Groups 1 and 2. In fact, all class I estimated statistics showed negative values, but they were statistically significant only for Group 2. This could depend on the fact that D*, F*, and D have low power to reject the constant size model and the power of such statistics rapidly decreases with increasing of the elapsed time since the expansion event, whereas Fs, which was statistically significant for both Groups 1 and 2, performs better (Ramos‐Onsins & Rozas, 2002). Additionally, all statistical class I tests used here increase the power to reject the constant size model with increasing the degree of expansion, and consequently, large samples are needed to detect small population growth events. This is consistent with the sample size for Group 1, smaller than for Group 2, and with results of the Bayesian analyses that indicated population growth for both Groups 1 and 2, but with different characteristics. In fact, Bayes factors supported the skyline model as best fit for Group 1, whereas exponential growth was preferred for Group 2. Interestingly, the slope of the skyline for Group 1 (Figure 4) was less steep than for Group 2, where it increased more abruptly, approximating an exponential model which was actually the preferred model for Group 2. Additionally, change in population size (1/2N_e_μ) was more marked in Group 2 than in Group 1. Thus, although both Groups 1 and 2 showed population growth, the degree of the demographic event was more pronounced in Group 2.

Also the mismatch analysis confirmed population growth for both Groups 1 and 2. In both cases, observed raggedness was lower than expected values under the constant size model. Given that raggedness has little power in detecting population growth (Ramos‐Onsins & Rozas, 2002), our results were conservative.

### 4.3 Dating demographic histories

Despite the degree of the demographic events was different in Groups 1 and 2, they seem to have initiated approximately at the same time, around 5E‐4 mutations/site. In this case, the application of the two rates provided by Crandall and collaborators would pinpoint the start of the demographic growth between approximately 77Kya (lower rate) and 9.4Kya (higher rate), both estimates suggesting that the event was recent. If the appropriate mutation rate is comprised between 5.3%/Myr and 0.65%/Myr, it is very likely that the demographic process may have started at the end of the Last Glacial Maximum (LGM), which is dated approximately between 19 and 20 Kya in the northern hemisphere and 14–15 Kya in the West Antarctic (Clark et al., 2009) and Central Pacific (Blard, Lave, Pik, Wagnon, & Bourles, 2007). In the tropics, glaciers could have reached their greatest extent 34Kya and were retreating approximately 21 Kya (Smith, Seltzer, Farber, Rodbell, & Finkel, 2005). Tropical Andes would have reached the LGM between 24.5 and 25.3 Kya, whereas deglaciation would have started 16.7–23.5 Kya, with some more recent deglaciations having occurred between 10 and 13Kya (Bromley et al., 2009; Shakun et al., 2015). If so, the proper μ is most likely more proximate to the higher value suggested by Crandall and collaborators and would correspond to approximately 5.0%–2.13%/Myr, given the time range of deglaciation events here considered. Slower μ would not be compatible with the resulting scenario, which would imply the absence of favorable environmental conditions for mangrove and ark mollusk expansion. In fact, compelling evidence demonstrated that late Pleistocene glacial climate originated well before 77Kya and persisted until the end of LGM (Huybers & Wunsch, 2005). The persistence of such conditions confined *Rhizophora* mangrove in glacial refugia until the species started expanding via dispersal from refugial areas at the end of LGM in the Caribbean Basin, Florida (Kennedy et al., 2016), Brazilian coasts (Pil et al., 2011), in the Gulf of California (Sandoval‐Castro et al., 2014), and in other areas for which no documented evidence exists. The expansion of mangroves would have favored the dispersal of *A. tuberculosa*. The contraction of geographical ranges during the glacial period, and then spatial and demographic population expansions when favorable conditions emerged during interglacial periods have been invoked to explain pattern of genetic variation is other bivalves inhabiting shallow waters (Xue et al., 2014).

## 5 CONCLUSIONS

In conclusion, we showed a high level of genetic variation in equatorial *A. tuberculosa*. Despite the potential long‐range larval dispersal, such variation is geographically structured with populations at north and south of the equator not being completely connected. As in other bivalves, a combination of life history traits and oceanic prevalent currents may explain the pattern observed. Climatic changes occurred at the end of the LGM may be responsible for the documented demographic expansions. While awaiting for more data becomes available from other locations, we suggest separate management of populations at north and south of the equator.

## CONFLICT OF INTEREST

The authors declare no competing interest.

## AUTHOR CONTRIBUTION

BD and VC designed the work; BD, KP, RA, and CC collected samples and contributed genetic data; GG and BD contributed data analysis; GG, BD, and VC drafted the manuscript. All authors critically revised and approved the final manuscript.

## Data Availability

The raw data underlying the main results of the study are archived in Genbank (accession numbers: MK043084 ‐ MK043325).
