# Supplementary figures and images for: Genetic structure, phylogeography, and demography of Anadara tuberculosa (Bivalvia) from East Pacific as revealed by mtDNA: Implications to conservation
Source: Ecol Evol. 2019 Apr 4;9(8):4392–402. doi: 10.1002/ece3.4937 (PMC6476791; doi:10.1002/ece3.4937)

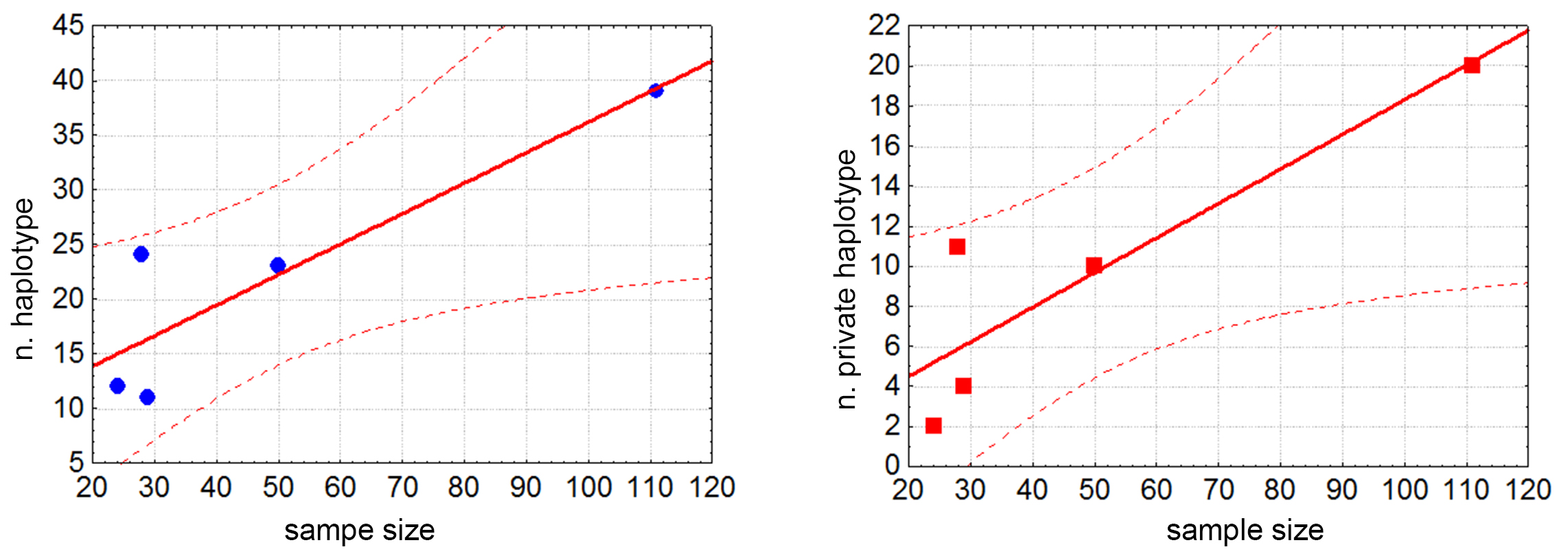

Supplement: Supplementary file 1 [file ECE3-9-4392-s001.tif]

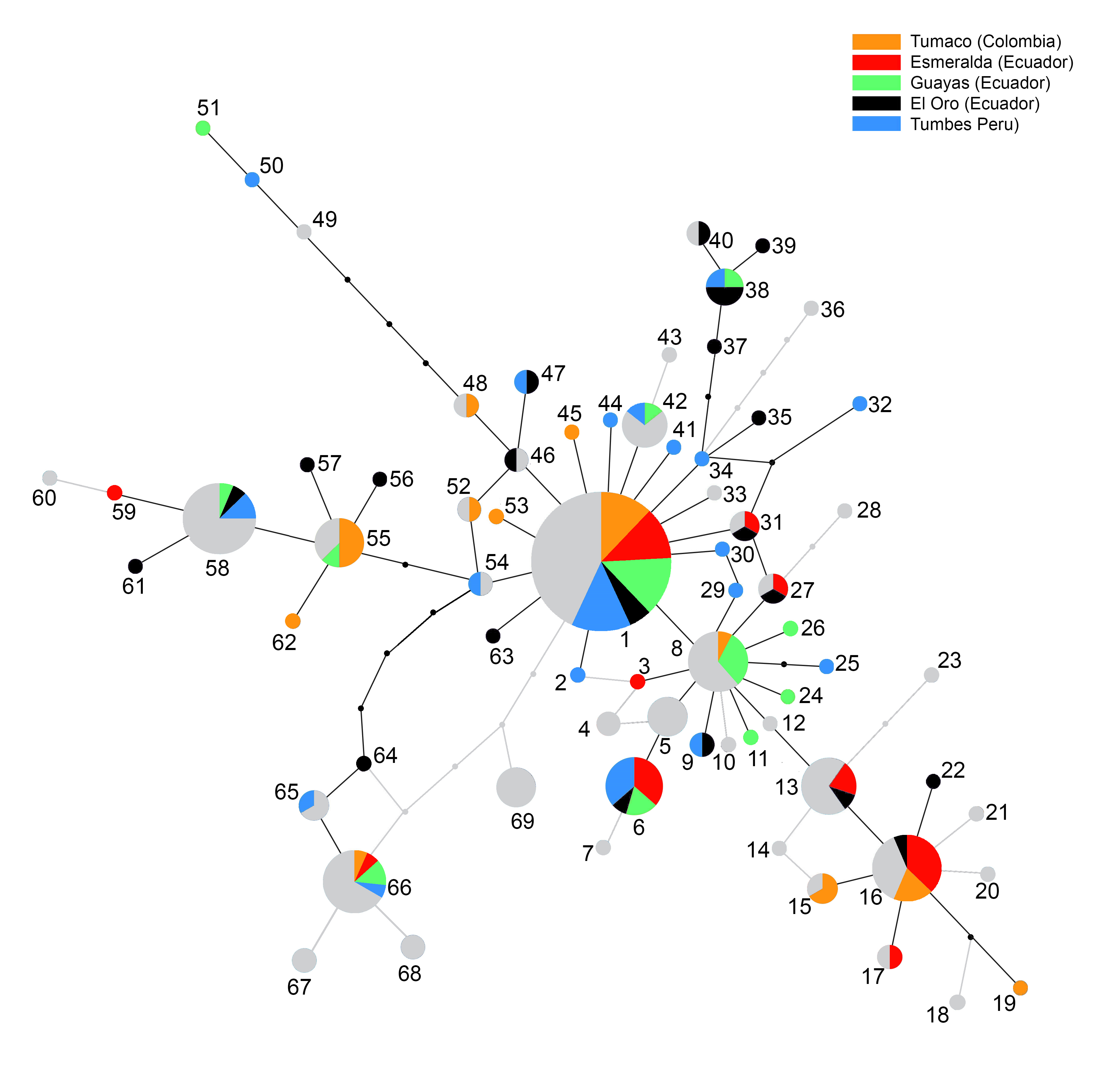

Supplement: Supplementary file 2 [file ECE3-9-4392-s002.tif]

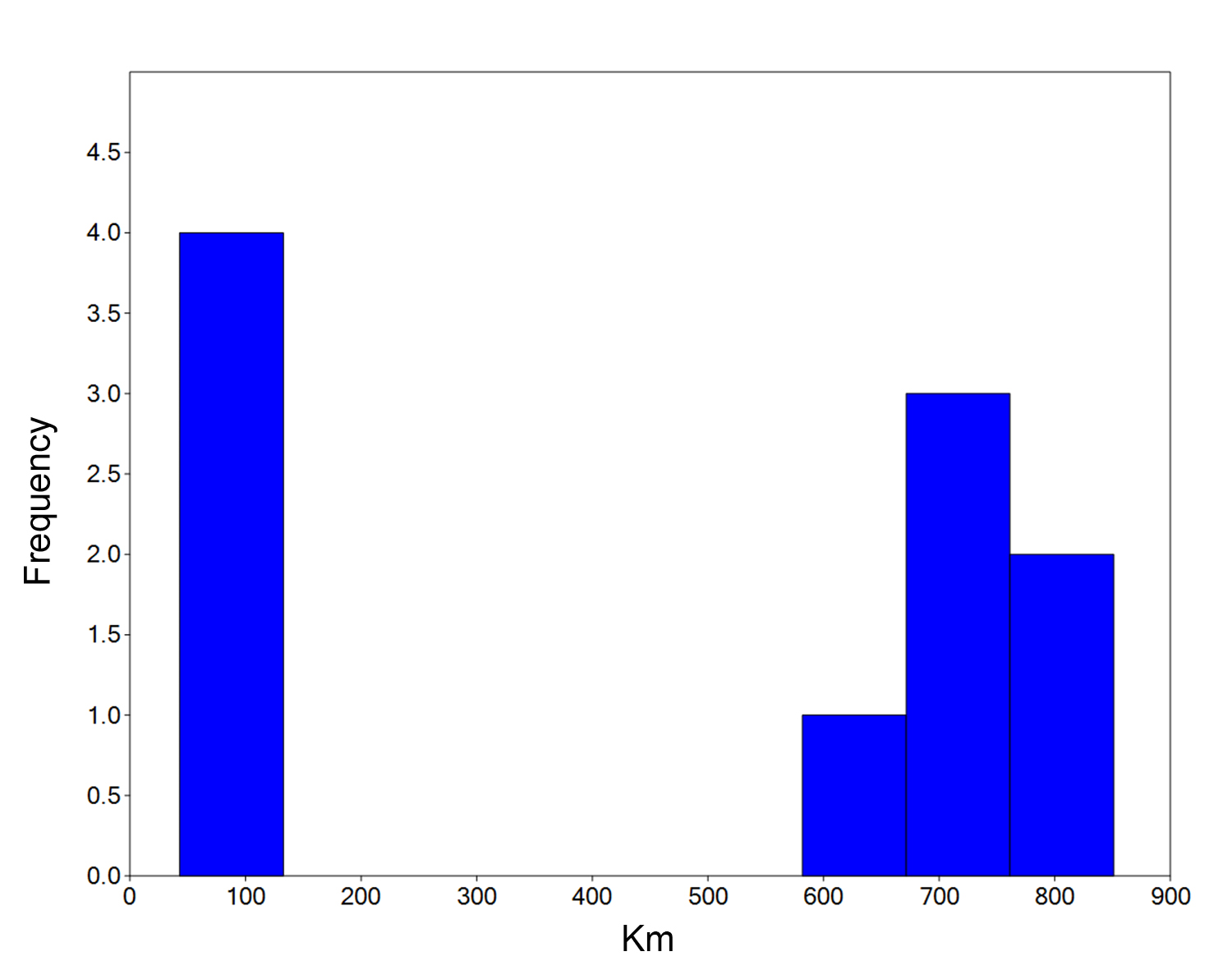

Supplement: Supplementary file 3 [file ECE3-9-4392-s003.tif]
