# Supplementary material for: Genetic structure, phylogeography, and demography of Anadara tuberculosa (Bivalvia) from East Pacific as revealed by mtDNA: Implications to conservation
Source: Ecol Evol. 2019 Apr 4;9(8):4392–402. doi: 10.1002/ece3.4937 (PMC6476791; doi:10.1002/ece3.4937)
Supplement: Supplementary file 4 [file ECE3-9-4392-s004.docx]

**SUPPLEMENTARY FIGURES LEGEND**

**Figure S1.** Left: linear regression between sample size (independent variable) and n. haplotypes (dependent variable). Right: linear regression between sample size (independent variable) and n. of private haplotypes (dependent variable).

**Figure S2.** Statistical parsimony network after reducing sample size. Grey color marks the reduction of haplotypes and branches respect to Fig. 2.

**Figure S3.** Distribution of pairwise geographic distances between sampling location. Geographic distances were calculated as the shortest pathways along the costal line.
